# Convention of Western Medical Schools

**Published:** 1849

**Authors:** 


### ARTICLE II.

### CONVENTION OF WESTERN MEDICAL SCHOOLS.

We see that the proposition for this Convention meets with favor generally, among the Medical Journals. but as yet we hear of no report from any of the schools directly. Of course, until there is some intimation from the schools, of their approbation of the project and willingness to send delegates, it is useless to appoint a time or place for meeting.

With Cincinnati as the place, and the last Tuesday in April next as the time, we would be entirely satisfied, provided all can be ready and an understanding had by that time, which we very much doubt.

That we may act understandingly in the matter, we propose that our friend, Professor Lawson, of the Western Lancet, act as Secretary of the project, and recommend that he privately address medical schools in the west, to ascertain whether they will all send delegates or not, and if there is a general concurrence, fix the time and give notice accordingly. Unless some general understanding can be had, it will be scarcely worth while for delegates to meet, as a partial representation cannot accomplish much.

